# Ceramic Laminate Veneers for Reestablishment of Esthetics in Case of Lateral Incisor Agenesis

**DOI:** 10.1155/2018/4764575

**Published:** 2018-01-17

**Authors:** Geórgia Silva, Ana Cristina Normandes, Edson Barros Júnior, Joyce Gatti, Kalena Maranhão, Ana Cássia Reis, Fernanda Jassé, Lucas Moura, Thaís Barros

**Affiliations:** ^1^School Superior of Amazonia (ESAMAZ), Belém, PA, Brazil; ^2^College São Leopoldo Mandic (SLMANDIC), Belém, PA, Brazil; ^3^School of Dentistry, School Superior of Amazonia (ESAMAZ), Belém, PA, Brazil

## Abstract

The increasing demand of patients looking for esthetics has resulted in the development of several techniques to restore anterior teeth. Conservative treatments should always be the first therapeutic option for the solution of aesthetic problems involving morphological changes and usually provide the result that the patient expects. In this context, ceramic laminate veneers, also known as “contact lenses,” are capable to provide an extremely faithful reproduction of natural teeth with great color stability and periodontal biocompatibility. Minimal or no preparation veneers are heavily advertised as the answer to patients' cosmetic needs, when properly indicated by the dentist. This paper reports a clinical case where lateral incisor agenesis was aesthetically corrected using ceramic laminates.

## 1. Introduction

Agenesis is defined as a numerical anomaly that expresses the lack of development of one or more teeth, occurring in approximately 25% of the population, and can affect deciduous and permanent dentitions resulting from a dental blade disorder which prevents the formation of the dental germ. It may also be referred to as partial anodony, hypodontia, or oligodontia [1–12].

In the Brazilian population, agenesis affects between 2% and 5% of people depending on the affected tooth and excluding the third molars (wisdom tooth) that ranges around 20% to 30%. According to Santos [13], the most frequently observed agenesis, excluding third molars, is the one that affects the maxillary lateral incisors (ILS) (37.1%), followed by the mandibular second premolars (32.26%) and maxillary second premolars (17.54%).

The esthetic and functional alteration that the agenesis of ILS can provoke is quite relevant, being a concern factor, not only for the patients with the anomaly but also for the dentists responsible for planning the case. This anomaly can generate a change between the dental arches; being an important factor predisposing to malocclusions, it alters the function of the stomatognathic system, besides causing a great aesthetic discomfort, which is the main complaint of the patient [1–12].

Several treatments are suggested in the literature in cases of the absence of one or more ILS. The options range from no treatment or even two possibilities of clinical interventions (1) to create adequate space for inclusion of the missing tooth/teeth [14] or (2) to close the available space in the dental arch, providing the contact of the central incisor with canine, associated to the reanatomization of the canine, transforming it into a lateral incisor [11, 15, 16].

The decision during the treatment planning implies in the identification of alternative procedures, the prediction of the relative probabilities in favor of the long-term desired result, and evaluation of the cost-risk-benefit relation of each alternative [11]. The decision should be understandable to the patient or caregivers and better meet the needs of the patient. Many challenges are involved in obtaining and maintaining optimal results [17–19].

With the evolution of restorative materials and adhesion procedures, ceramic laminates have been used in corrections and dental reconstructions with a high predictability of success, especially because they require less wear or, in many cases, no wear, preserving a greater amount of sound dental structure, contributing to pulp and periodontal health [20, 21]. Besides these advantages, the aesthetic treatment using ceramic laminates also presents other ones such as biocompatibility, color stability, and good optical properties, enabling the dental reestablishment with biomechanical characteristics similar to natural teeth [22–25].

The proper selection of a ceramic system for certain clinical situations may provide greater longevity of these restorations. Although most of these systems promote good esthetic results, some are better suited for anterior regions because of the greater translucency of the material. Several criteria can be used by the professional to select the most appropriate ceramic system, such as esthetics, marginal adaptation, biocompatibility, resistance, cost, and ease of manufacturing [26–32].

Therefore, the objective of this work is to describe the esthetic treatment of a patient affected by ILS agenesis (12 and 22), by means of ceramic laminate veneers.

## 2. Diagnosis and Etiology

### 2.1. Clinical Case

**Figure 1:**
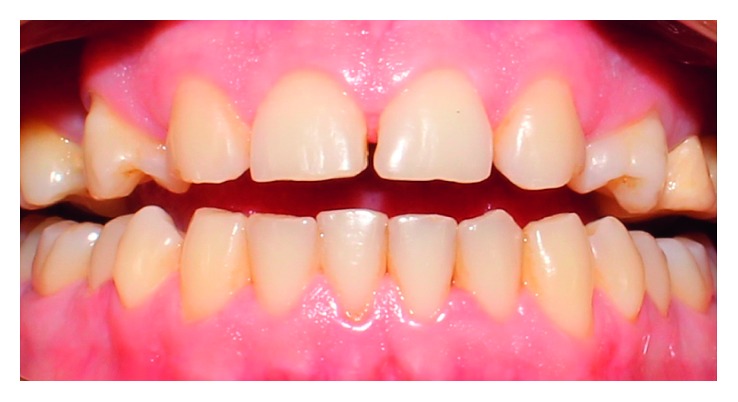
Preoperative view of the patient's smile.

A 28-year-old male patient was concerned about the esthetic of his smile. After the anamnesis and clinical and radiographic examinations, it was verified that the patient presented agenesis of dental elements 12 and 22; elements 13 and 23 occupied the lateral incisors' space; and elements 14 and 24 were rotated (Figure 1).

#### 2.1.1. Treatment Plan

After case evaluation by the clinician, the first option of a treatment plan proposed to the patient was correction of the positioning of the canines and premolars by means of orthodontic movement and, later, implantation of the lateral incisors. However, this option was not accepted by the patient who was not interested in undergoing the surgical procedure.

The second treatment planning option proposed was reanatomization of elements 14, 13, 11, 21, 23, and 24, by means of the preparation of ceramic laminates in order to better harmonize the patient's smile. All the advantages and disadvantages of the treatment were exhaustively explained to the patient who, after understanding the proposed treatment, agreed with the execution of the procedure.

#### 2.1.2. Diagnostic Waxing

The first clinical step of the treatment was to perform molding using condensation silicone Zetaplus (Zhermack) to study the case. After the model was obtained, elements 13 and 23 were waxed so that the anatomy was as close as possible to the anatomy of upper lateral incisors. The elements 14 and 24 had their rotation “corrected” and received anatomical characteristics of canines.

**Figure 2:**
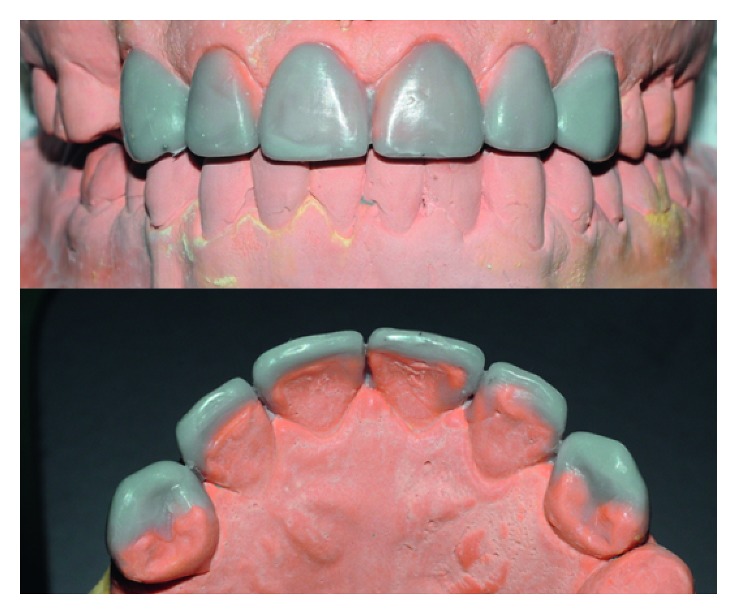
Diagnostic waxing proposed to reanatomize and provide harmony to the patient's smile.

According to the principles of proportionality and in order to maintain the smile harmony, elements 14, 13, 11, 21, 23, and 24 were enlarged both in the mesiodistal and in the cervicoincisal dimensions.  Figure 2 shows the proposed waxing model.

#### 2.1.3. Tooth Whitening and Mock-Up

**Figure 3:**
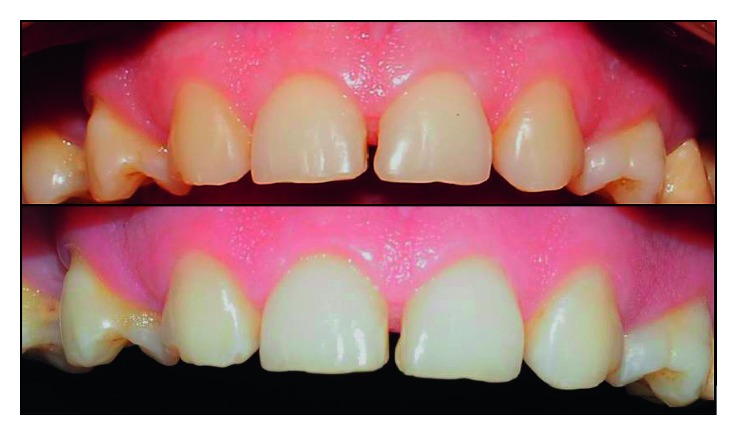
Result obtained after bleaching.

In order to optimize the aesthetic result of the case, the patient underwent an in-office whitening session. Three 15-minute applications of Whiteness HP 35% (FGM) were performed. No sensitivity was reported by the patient, and the result obtained (Figure 3) was considered satisfactory (Initial color: color A3/Final color: A2-VITA Classical scale).

After 7 days, the mock-up was performed so that the patient could visualize the simulation of the proposed treatment, as well as to allow us to detect the need for corrections in the diagnostic waxing.

**Figure 4:**
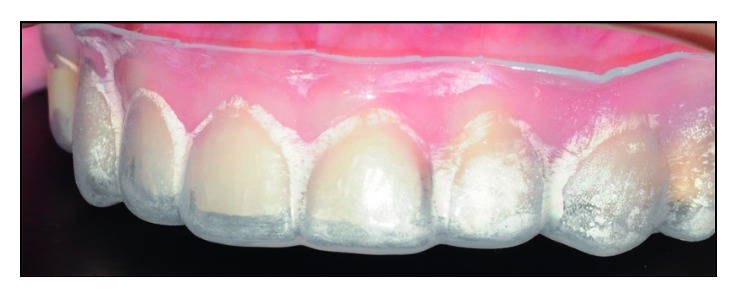
Silicone tray in position.

The waxed model was duplicated, and a silicone tray was made using a vacuum plasticizer, which allowed to “transfer” the proposed waxing to the patient's mouth (Figure 4).

**Figure 5:**
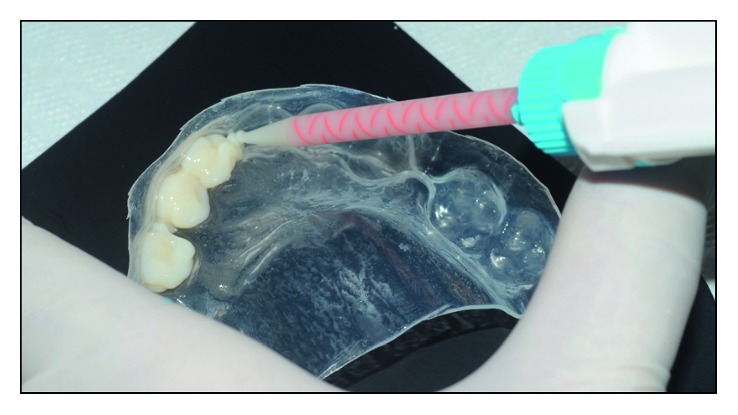
Silicone tray being loaded with Protemp 4 (3M ESPE).

A bisacrylic resin (Protemp 4-3M ESPE) was used to simulate the proposed aesthetic resolution. The tray was loaded with the material (Figure 5) and taken into position after 40 seconds from the start of loading.

**Figure 6:**
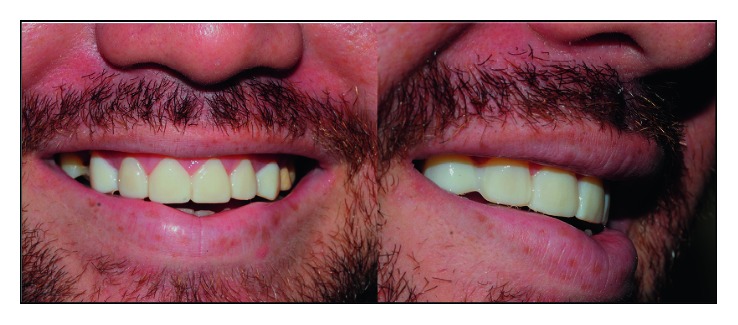
Result obtained with mock-up.

After 1 minute, the tray was removed. The “mock-up” was finished with a 15C scalpel blade, 3195 F and FF diamond burs and abrasive papers.  Figure 6 illustrates the result obtained after mock-up.

The patient was extremely satisfied with the result of the simulation; however, clinically, a small overconfiguration was observed in the mesial region of element 24. The overconfiguration was corrected in the waxed model so that the patient did not present any periodontal changes after finishing of treatment.

#### 2.1.4. Selective Wear

After approval by the patient and adjustments made in the diagnostic waxing, the phase of selective wear was started. It was decided to perform these abrasions only in the areas that presented a thin layer of wax in the waxed model, thus guaranteeing a satisfactory thickness in the future ceramic laminate.

**Figure 7:**
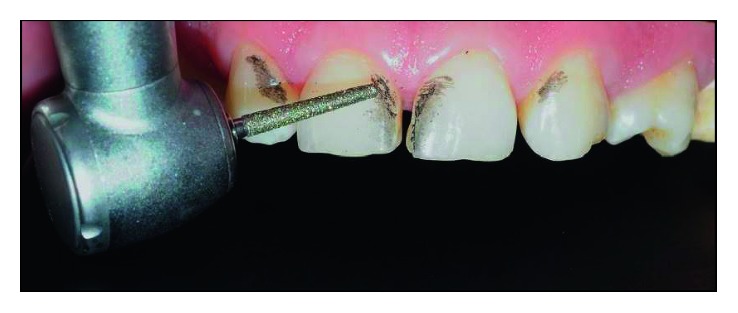
Regions selected to receive selective wear.

The regions that received selective wear were cervical-mesial of element 13, vestibular-mesial of elements 11 and 21, and cervical-mesial of 23, as shown in Figure 7.

**Figure 8:**
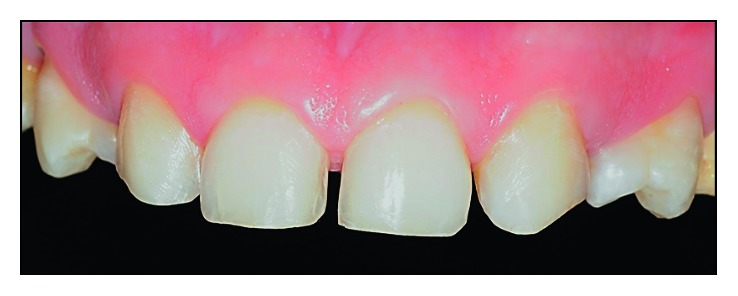
Clinical situation after finishing the wear and polishing steps.

All wear was performed using the 4138F (Poul Sorense) diamond bur and polished with Optimize (TDV) abrasive rubbers and Polimax Felt Disc (TDV). No cervical termination region was established.  Figure 8 shows the clinical situation after finishing the wear and polishing steps.

#### 2.1.5. Impressions and Color Selection

**Figure 9:**
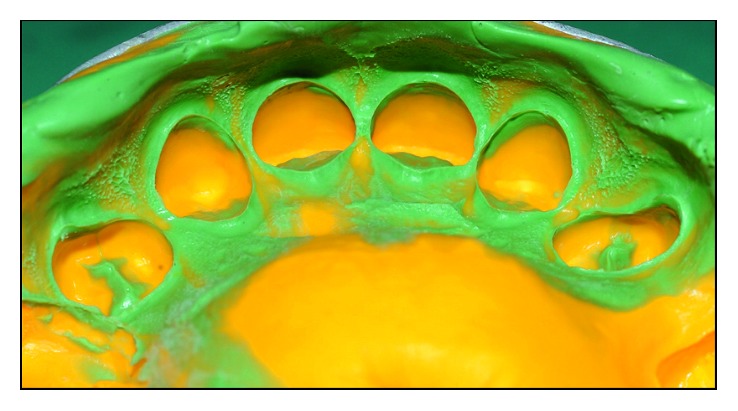
Impression of preparations made with addition silicone (Express-3M ESPE).

In sequence, the impression of preparations was performed using addition silicone (Express-3M ESPE) by means of the double mixing technique and without gingival clearance (Figure 9). The antagonistic arch was molden using alginate (Hydrogun5-Zhermarck), and an interocclusal record was taken using a wax plate 7. Color 1M2 was selected using the VITA 3D Master scale. The laboratory technician was informed that the “preparation” was all in enamel.

#### 2.1.6. Laboratory Steps

**Figure 10:**
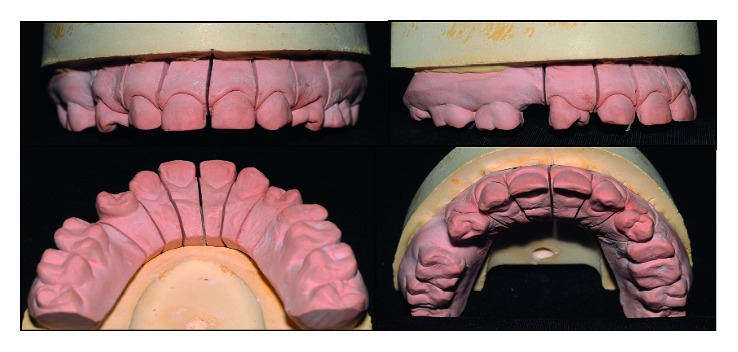
Cutting of the plaster work model.

**Figure 11:**
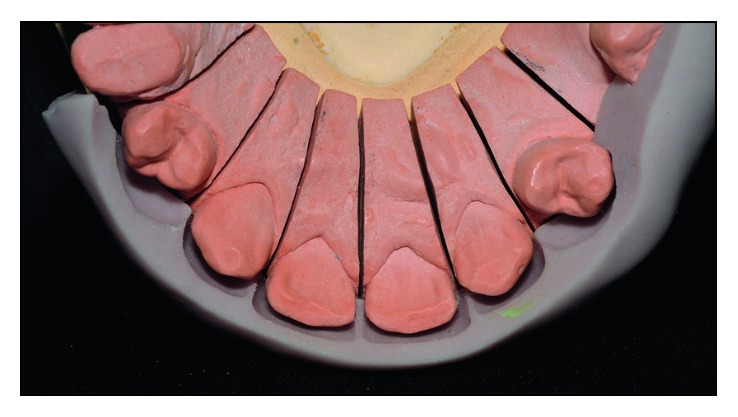
Silicone wall in position. The thicknesses of the ceramic laminates can be observed.

The work model was screwed up to obtain a better adaptation of the laminates on the proximal faces (Figure 10). A silicone wall (Zetalabor-Zhemarck) was made based on the waxed model, in order to allow a better visualization of the thickness of the prosthetic pieces (Figure 11).

**Figure 12:**
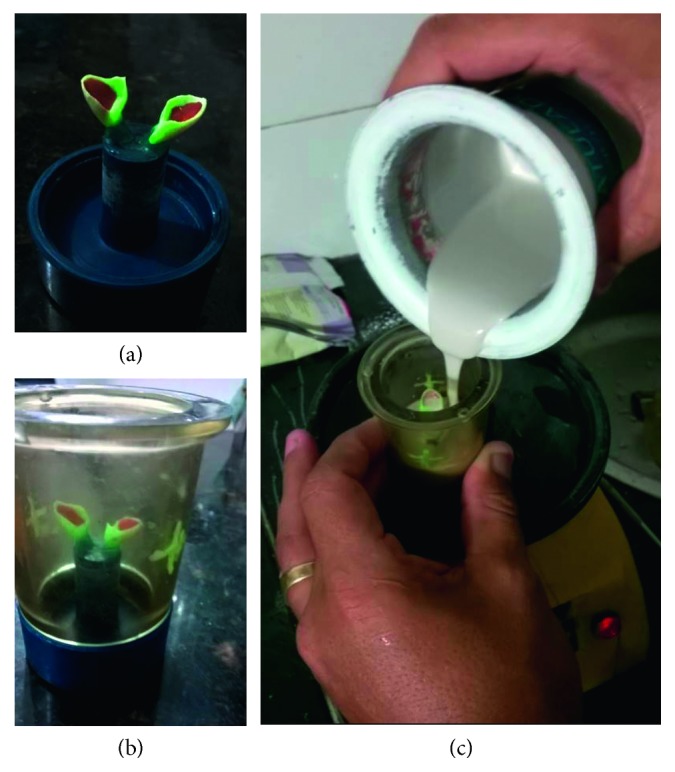
Laboratory steps for manufacturing of ceramic laminates. (a) Fixation of the waxed veneers at the base, (b) adaptation of the silicone ring at the base, and (c) addition of the coating liquid.

The porcelain used was the VITA PM-9, which is a feldspathic pottery reinforced by Leucita. The laminates are made by the lost wax technique, in which the ceramic is injected under high temperature (1000°C) and pressure (4, 7 bar) in a coating mold.  Figure 12 shows the steps of lost of wax (a), positioning of the mold (b), and inclusion in the coating material (c).

**Figure 13:**
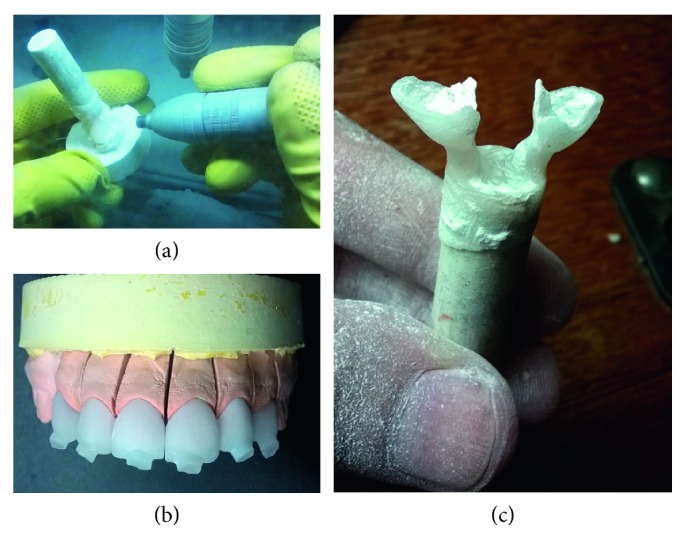
(a) Removal of the laminates from the coating mold using shot blasting with glass microspheres; (b) ceramic pieces placed in the plaster model, still with the remnants of the feed channels; (c) view of the injected parts attached to feed channels.

To remove the newly injected ceramic from the coating mold, blasting with glass beads with a 50 μm granulation and a pressure of 2 bar (Figure 13) was used. After the coating is completely removed, the injection channel is separated with a diamond disc, followed by adjustments, finishing, and polishing (Figures 13 and 13).

#### 2.1.7. Cementation

**Figure 14:**
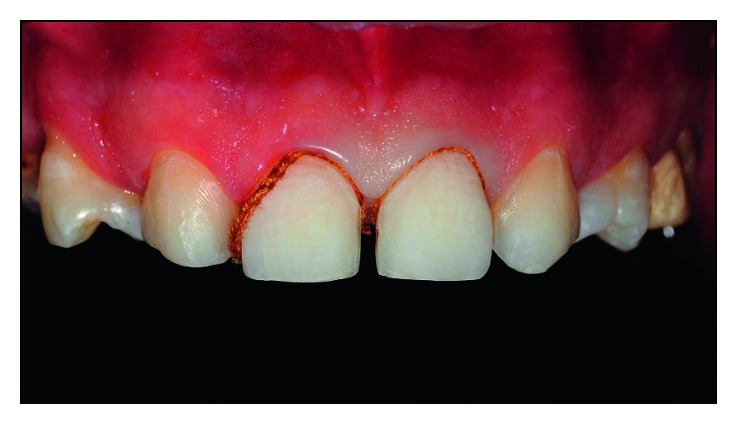
Gingival clearance prior to cementation.

The ceramic laminates were tested on the patient to check and adjust the proximal contact points. For cementation, a photoactivated resin cement (AllCem Veneer-FGM) was used, which has a try-in system—it is a color proof paste that mimics the colors of the resin cement after light curing. The selected color for the cement was A3. The adhesive protocol was minutely performed in the dental structure: conditioning with 37% phosphoric acid (Condact 37-FGM) for 30 s, followed by washing for 60 s and removal of moisture. After performing this step, gingival clearance (Figure 14) using retractor wire (Retractor # 00-FGM) was performed to ensure that there is no excess adhesive and/or cement in the gingival sulcus region. Then the adhesive system (Adper SingleBond-3M ESPE) followed by photopolymerization for 10 s were applied.

The inner surfaces of ceramic laminates were conditioned with 10% hydrofluoric acid (Condac 10%-FGM) for 20 s, followed by rinsing with copious amounts of water, drying, and, afterwards, the application of three layers of silane (Prosil-FGM). After silane evaporation, the adhesive system Adper Single Bond 2 (3M ESPE) was applied.

**Figure 15:**
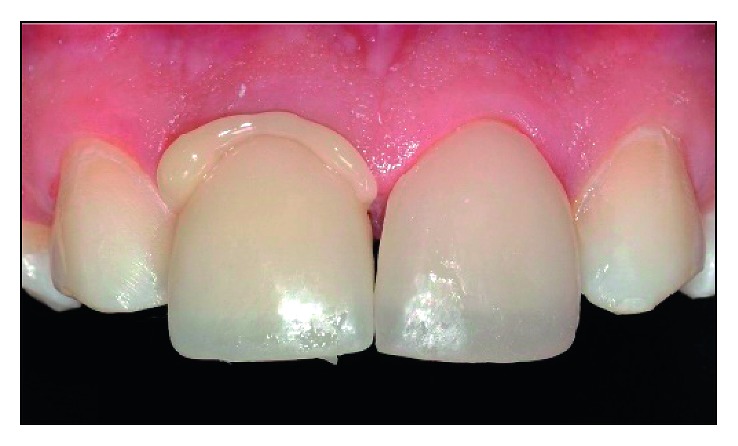
Extravasation of the cementing agent in element 11.

The cement was applied on the cementation surfaces of ceramic laminates. After that, veneers were positioned with cement until its excess had overflowed (Figure 15).

**Figure 16:**
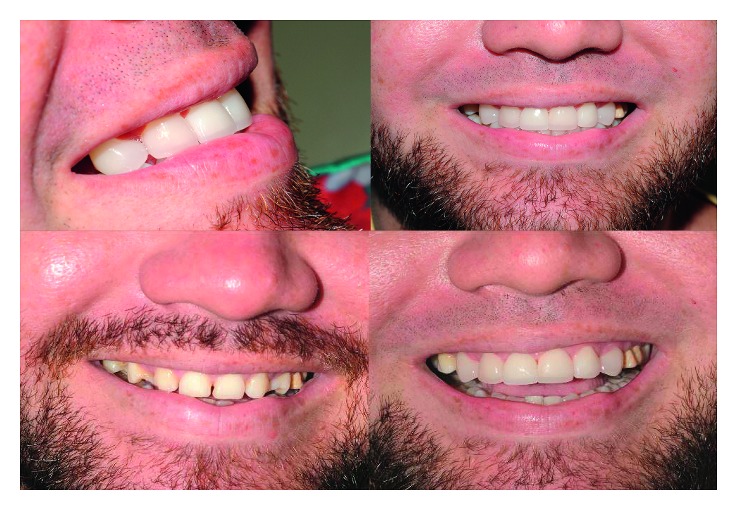
Final result.

The cementation sequence adopted was upper central incisors, followed by the premolars and, finally, the canines. After the cementation, the finishing steps were accomplished with fine-grained and extrafine diamond burs, followed by polishing with abrasive rubbers (Exa-cerapol-Edenta). The final result obtained is illustrated in Figure 16.

## 3. Discussion

Aesthetics has been increasingly required in today's dentistry. With the increase of access to information, through the Internet, books, and magazines, the population becomes more and more demanding.

Therefore, aesthetics should be closely associated with the patient's wishes, respecting the principles of smile harmony, oral rehabilitation, correct diagnosis, treatment plan, and the type of material to be used in the selected treatment.

In the Brazilian population, the prevalence of dental agenesis varies in percentage, depending on the study. The values found were 29.5%, 7.9%, and 2.9% in which agenesis of the third molar is the most common [13]. Opinions vary on the second most commonly affected tooth; some studies show that the second lower premolar has the highest in prevalence while others show that the superior lateral incisor has the highest [1, 3, 5, 13].

The treatment of dental anomalies is always a challenge for the general practitioner. For Alavi et al. [25] and Giordano [28], the best method would be the use of composite resin owing to the preservation of dental structure and the financial cost. For Nathanson and Riis [26], Giordano [28], Van Dijken [29], and Höland et al. [30], ceramic restorative materials would be the best choice since composite restorations undergo time action and require regular maintenance. In addition, composite resins exhibit lower clinical longevity due to greater susceptibility to pigmentation and marginal fractures [33].

In this clinical report, the use of ceramic laminates was the treatment of choice, in particular, the ceramic system VITA PM-9 because this material presents the following advantages: ability to reproduce the appearance of natural teeth, good translucency, excellent resistance, and similar biomechanical behavior to the tooth structure.

Nowadays the treatment with ceramic laminates rehabilitation is in use in a large scale; this is mainly due to being a very conservative treatment, where the wear of the dental element is minimal preserving the dental structure, especially in young patients [22–32]. However, because they are relatively recent techniques, they still do not have a common sense, mainly regarding their indications and limitations [3, 4, 11].

Machry [34] affirms that one must always keep in mind the correct diagnosis to carry out a correct planning and, consequently, an appropriate treatment sequence for each case. Although recent, clinical evaluations have shown a very promising outlook, today they represent an alternative that the clinician needs to employ in selected cases.

## 4. Conclusion

The diversity of ceramic systems currently available in the world market is due to the increasing search for aesthetic excellence. The systems present advantages and disadvantages when compared to each other.

The most important in clinical cases is to establish a careful and realistic treatment plan, taking into account the patient's wishes. The time factor is often determinant for the selection of the treatment plan, since some patients want to solve their problem in the shortest time possible.

Thus, it can be concluded that the performed procedure corresponded very well with expectations of the patient and that the techniques used to do so were well performed, and it was obtained a satisfactory result.
